# Kinetic analysis of effects of temperature and time on the regulation of venom expression in *Bungarus multicinctus*

**DOI:** 10.1038/s41598-020-70565-2

**Published:** 2020-08-24

**Authors:** Xianmei Yin, Shuai Guo, Jihai Gao, Lu Luo, Xuejiao Liao, Mingqian Li, He Su, Zhihai Huang, Jiang Xu, Jin Pei, Shilin Chen

**Affiliations:** 1 Key Laboratory of Distinctive Chinese Medicine Resources in Southwest China, Pharmacy College, Chengdu University of Traditional Chinese Medicine, Chengdu, 611137 People’s Republic of China; 2 Key Laboratory of Beijing for Identification and Safety Evaluation of Chinese Medicine, Institution of Chinese Materia Medica, China Academy of Chinese Medical Sciences, Beijing, 100700 China; 3 Cancer Institute of Integrated Traditional Chinese and Western Medicine, Zhejiang Academy of Traditional Chinese Medicine, Tongde Hospital of Zhejiang Province, Hangzhou, 310012 Zhejiang China; 4 The Second Clinical College, Guangzhou University of Chinese Medicine, Guangzhou, 510006 China

**Keywords:** Gene expression analysis, Sequencing

## Abstract

Venom gland is a highly efficient venom production system to maintain their predatory arsenal. Venom toxins mRNA has been shown to increase abruptly in snake after venom expenditure, while the dynamics of venom accumulation during synthesis are poorly understood. Here, PacBio long-read sequencing, Illumina RNA sequencing (RNA-seq), and label-free proteome quantification were used to investigate the composition landscape and time- and temperature-dependent dynamics changes of the *Bungarus multicinctus* venom gland system. Transcriptome data (19.5223 Gb) from six adult *B. multicinctus* tissues were sequenced using PacBio RS II to generate a reference assembly, and average 7.28 Gb of clean RNA-seq data was obtained from venom glands by Illumina sequencing. Differentially expressed genes (DEGs) mainly were protein processing rather than venom toxins. Post-translational modifications provided the evidence of the significantly different proportions of toxins in the venom proteome with the changing of replenishment time and temperature, but constant of venom toxins mRNA in the venom gland transcriptome. Dynamic of toxins and genes involved in venom gland contraction suggesting the formation of the mature venom gland system would take at least 9 days. In addition, 59 toxin processing genes were identified, peptidylprolyl isomerase B of which underwent positive selection in Toxicofera*.* These results provide a reference for determining the extraction time of venom, production of polyclonal and monoclonal antibody for precise treatment plans of venomous snakebites, and construction of an in vitro synthesis system for snake venom protein.

## Introduction

Snakebite envenoming is a neglected tropical disease, which harms more than 400,000 people and kills more than 100,000 every year^1^. But developing an efficacious and safe snakebite treatment remains a challenge for the research community. The first requirement for the development of snake venom drugs is to understand the composition of snake venom. However, snake venom proteins show high levels of variation with changes in environment^2^, prey^3^, and replenishment stage^4^. Although exhaustion of venom supply probably never occurs under natural conditions^5^, it is likely that venom supply decreases upon envenomation of multiple prey items or an intense struggle with a predator. Much research has focused on the diversity of snake venom with respect to regeneration time^6–9,10^, implying the possibility of variations in whole-venom composition, depending on the stage of replenishment. Thus, understanding the timing of replenishment is a significant step in learning how venom supply might influence venom composition and activity. Also, understanding the rate of venom replenishment is important for researchers wishing to further study the biochemistry of snake venom. In addition, sudden upregulation of RNA synthesis in venom gland tissue has generally been shown to follow with a rapid synthesis of snake venom^9,10^, leading to increased interest in the proteins involved in snake venom synthesis.

*Bungarus multicinctus* is among the most venomous land snakes in the world according to its median lethal dose (0.09–0.108 mg/kg)^11,12^, and pose a serious medical problem in tropical and subtropical countries^13^. In addition, they became an important farmed species in china because of high medicinal and economic value, and have therefore attracted considerable research attention. Their venom compositions have been studied intensively over the past decades^14–17^. However, there have been no reports of the time- and temperature-dependent dynamic changes of the venom system in *B. multicinctus*, although temperature is an important environmental factor in the regulation of snake venom composition. Moreover, although early studies pay attention to the total RNA and total protein levels respectively^18^, little is known about the expression dynamics of individual venom components and other important parts of involving venom synthesis.

In addition, traditional approach for isolating individual toxins was not possible to characterize venom as a whole system and determine its multiple effects on an entire organism. The emergence of omics, bioinformatics, and computational biology has provided new tools that have already been successfully applied to venom research^19,20^. For instance, PacBio full-length transcription can be used to obtain organism’s genetic information, combined with high-throughput RNA sequencing (RNA-seq) for gene expression quantification. Here, we integrated three complementary high-throughput techniques, PacBio long-read sequencing, Illumina RNA-seq, and proteome label-free quantification, to systematically explore the composition landscape and time- and temperature-dependent dynamic changes of the venom gland system in *B. multicinctus*, in order to provide data support for studies of snake venom regulation and envenomation.

## Results

### Construction of PacBio full-length transcriptome

Two pooled samples representing polyA RNA from six tissues (muscle, kidney, lung, liver, heart, venom gland) of *B. multicinctus* adults were sequenced to obtain wide coverage of the *B. multicinctus* transcriptome. After filtering out low-quality data, the adapter and primer sequences of the reads were truncated and 19.5223 Gb of clean data were obtained. As shown in Fig. S1, raw data yielded 397,370 and 498,609 circular consensus sequences (CCSs), respectively. All CCSs were further classified into full-length (FL), non-FL (nFL), and FL non-chimeric (FLNC) transcripts (Fig. S2). To improve the accuracy of the isoforms, the low-quality transcripts were corrected with the corresponding Illumina sequencing data using LSC software. The full-length transcript length distribution after correction is shown in Fig. S3. Furthermore, 162,660 consensus isoforms were obtained, with a mean read length of 1991 bp, after the two libraries were merged and redundancies were removed. Finally, after eliminated transcripts having identical intronic coordinates with reference annotations, total 53,363 unigenes was obtained and 4,141 unigenes has not been annotated (Table 1).

**Table 1:** Summary of sequencing reads after correction.

Isoforms	Min length	Mean length	Median length	Max length	N50	N70	N90	Total nucleotides	Unigenes
162,660	54	1,991	1,856	7,116	2,461	1,921	1,256	323,906,399	53,363

### Illumina sequencing and expression quantification

Venom was extracted from *B. multicinctus* at four time points (days 3, 6, 9, and 12) and three temperatures (15, 28, and 32 °C) using three biological replicates (B15-3-1 to B32-12-3; Table S1). Time point selection was based on the previous study of replenishment time in elapid and viperid^6–9^, and temperature depended on the optimum breeding temp (15–30 °C)^21^. Two extreme temperature and a intermediate temperature was selected to investigate dynamic change of venom gland. No venom was extracted from the animals for at least 25 days prior to the start of the study. The first venom extraction was referred to as mature venom (B0-0-1 to B0-0-3). Some samples were excluded due to dying for staying in a high-temperature environment for too long. In total, 29 venom gland samples were sequenced using the Illumina platform. The average data volume of a sample was 7.28 Gb data. The rates of Q20 values of all samples surpassed 97.5% (Table S1), which indicated that the sequencing quality satisfied the needs of the subsequent analysis. Additionally, sequencing report shown no AT or GC separations, suggesting that the sequencing was stable (Fig. S4). Clean reads of every sample were aligned to the PacBio sequence respectively, the average aligned ratio of each sample was 69.9% (Table S1), indicating that the clean reads data of every sample were comparable among. Therefore, the results of transcriptome sequencing were available.

### Analysis of differentially expressed genes (DEGs)

There were significant difference among replenishment time and temperature, with average 462 unigenes upregulated and 520 downregulated (Figs. S5, S6, and S7). Following the identification of DEGs, we analyzed their functions by classifying and enriching their gene ontology (GO) functions. Detailed proportions of the GO annotations for DEGs are shown in Figs. S8, S9, and S10, indicating that molecular functions, biological processes, and cellular components were well represented. The GO functional classification showed that DEGs were mainly enriched in 46 GO terms. The biological functions of the DEGs from the venom gland of *B. multicinctus* were enriched in cell stimulation responding to temperature and regeneration stage. We mapped the DEGs to the reference canonical pathways in the Kyoto Encyclopedia of Genes and Genomes (KEGG) database to further identify the differences in active metabolic pathways for different temperatures and regeneration stages (Figs. S11 and S12). The pathway and the top ten DEG enrichments are shown in Table S2. For genes from the venom gland of *B. multicinctus*, temperature and regeneration stage caused changes in signal transduction, global and overview maps, cancers, infectious diseases, immune system, transport and catabolism, folding, sorting and degradation, and translation.

### Comparison of transcriptome and proteome of mature venom

**Figure 1:**
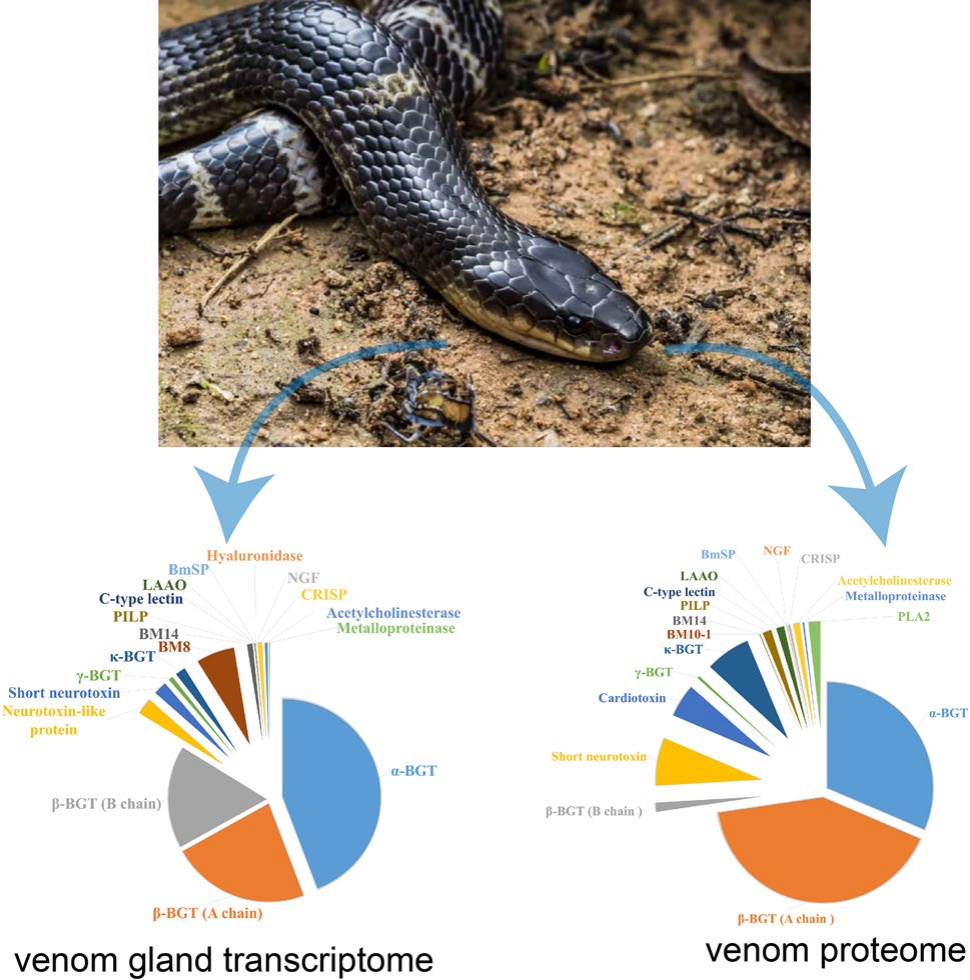
Distribution of toxins in the mature venom gland transcriptomes and venom proteomes of *Bungarus multicinctus*.

Next, we investigated the transcriptome (fragments per kilobase million (FPKM) metrics) and proteome [label-free quantification (LFQ)] of venom from the mature venom gland of specimens. The venom gland transcriptome derived from our 29 *B. multicinctus* individuals comprised a total of 22,316 unique non-toxin transcripts and 46 unique toxin transcripts. Similar to privious study, the most abundant toxin transcripts in *B. multicinctus* were those for 3FTxs, β-BGT, C-type lectin, L-amino-acid oxidase, venom nerve growth factor, cysteine-rich venom protein, acetylcholinesterase, metalloproteinase, hyaluronidase, and snake venom serine protease. These toxins constituted > 98% of venom gland transcripts (Fig. S13). The 3FTxs and β-BGT family toxins made up the majority of *B. multicinctus* toxins, accounting for more than 98% of toxin transcripts (Fig. 1). At the protein level, 43 venom proteins belonging to 8 different protein families were identified (Table S3). The proteome had similar toxin composition to the transcriptome (Fig. 1). The majority of differences were found in low-expressed toxins. Of course, we cannot exclude the possibility of annotation errors for trace-expressed proteins. However, the proportions of toxin proteins were different from those of toxin gene expressions. The proportion of α-BGT mRNA was significantly higher than that of protein, yet κ-BGT gene expression was lower than that of protein (Fig. 1). The expression levels of the gene encoding the A chain of β-BGT were much the same as those of the B chain, yet B chain protein levels were significantly lower than those of the A chain. These results indicate that toxin proteins may undergo post-translational modifications.

### Comparison between transcriptome and proteome during venom replenishment stages at different temperatures

**Figure 2:**
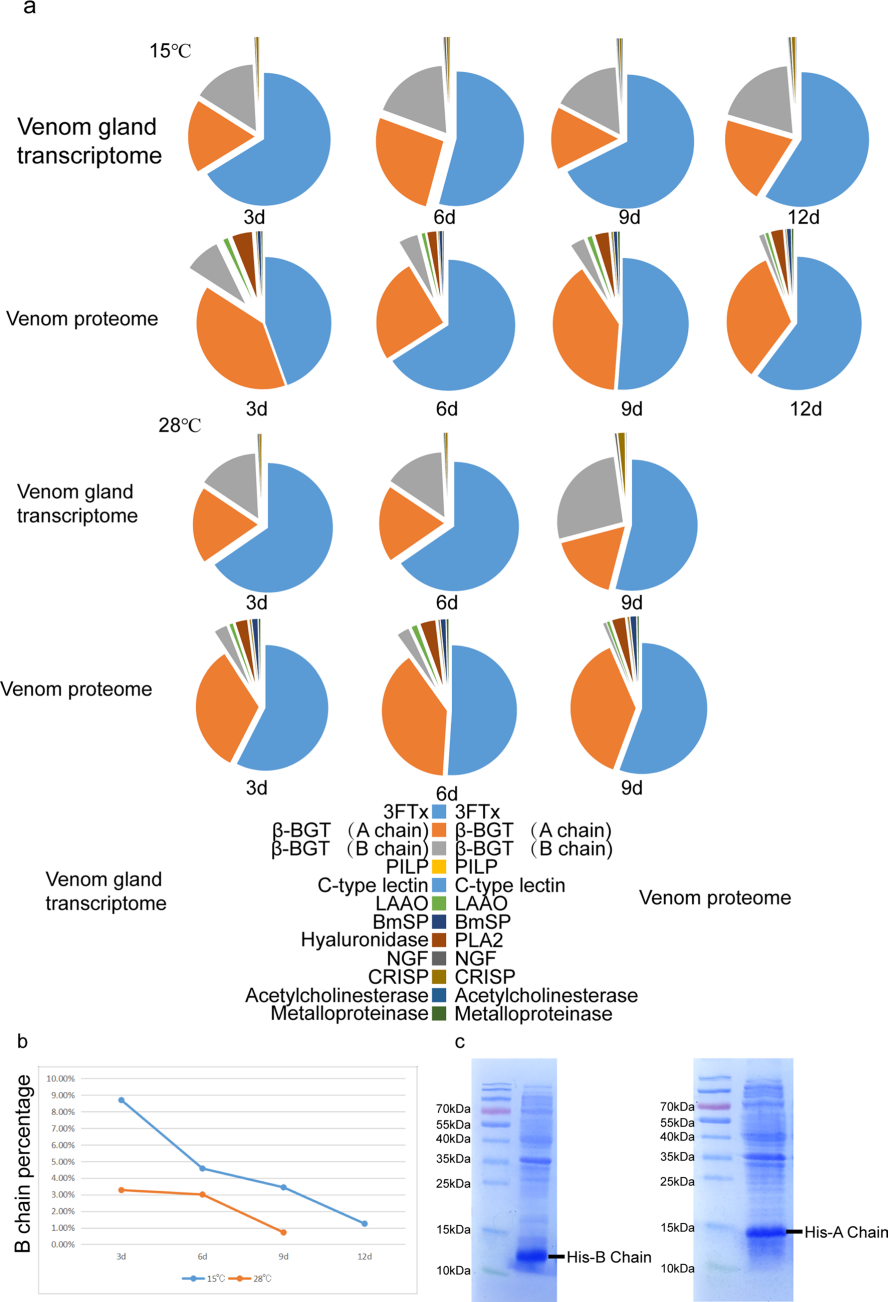
Distribution of toxins in the venom gland transcriptomes and venom proteomes of *B. multicinctus* at different replenishment times and temperatures. **(a)** Venom was extracted at four time points, referred to as day 3 (3d), day 6 (6d), day 9 (9d), and day 12 (12 d) and two temperature: 15 ℃ and 28 ℃ respectively. 28 ℃, day 12 and some 32 ℃ snakes was died due to staying in high temperature environment for too long time. Distribution of toxins in the venom gland transcriptomes and venom proteomes of B. multicinctus at 32 ℃ see supplementary Fig. 14. **(b)** The proportion of venom B chain of β-BGT in the venom proteome at different replenishment times and temperatures. (c) SDS-PAGE show the size of His6-B chain and His6-A chain expressed in *E. coli,* original gels figure are presented in Supplementary Fig. S15, the cDNA sequence and protein molecular weight of A Chain and B chain was show in supplementary Table 4.

**Figure 3:**
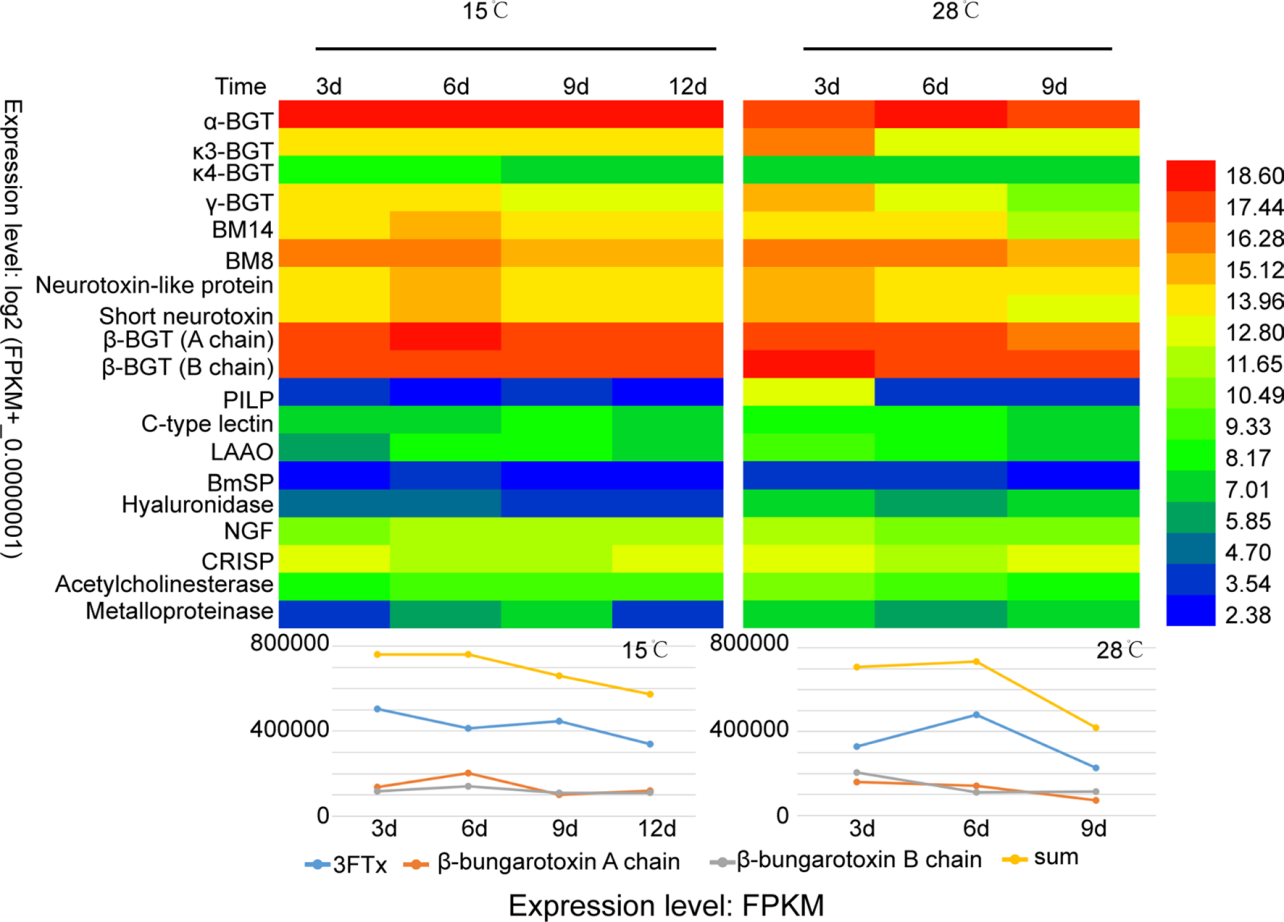
Expression dynamics of toxins in the venom gland of *Bungarus multicinctus*. **(A)** Heatmap representation of the expression analysis of toxin genes at different replenishment stages and temperatures. **(B)** Expression dynamics of three main to xin family genes (3FTxs, A chain of β-BGT and B chain of β-BGT) and total toxins.

The composition and relative proportions of venom mRNA appeared to remain constant over the time-course of venom replenishment (Fig. 2a). This was consistent with a previous study showing that mRNA is a remarkably stable component of venom^4^. Intriguingly, the expression ratio of the genes encoding the A and B chains of beta-BGT remained almost 1:1 during replenishment at 15 °C or 28 °C (Fig. 3). Individual toxin gene expression profiles over time and temperature varied slightly after venom expulsion. Overall, the expression levels of toxin genes were higher from days 3–6 (Fig. 3) than at any other time of the venom re-synthesis cycle. The mRNA expression of snake venom 3FTx peaked on day 3 when cultured at 15 °C but on day 6 at 28 °C. The mRNA expression levels of the A and B chains of β-BGT peaked day 6 at 15 °C but on day 3 at 28 °C. However, the total RNA of venom peaked on day 6 at both 15 °C and 28 °C post venom expulsion and decreased afterwards. In contrast to the venom gland expression data, the proportion of toxin proteins in the venom proteome varied significantly at different replenishment stages and temperatures. In particular, the proportion of β-BGT B chain protein decreased gradually with replenishment time (Fig. 2b). The B chain of β-BGT readily formed a stable polymer in vitro (the protein molecular weight of the B chain expressed in vitro was nearly twice that found in nature), yet this was not the case for the A chain (Fig. 2c and Fig. s15). Therefore, the proportions of A and B chain proteins varied significantly with time of replenishment, possibly owing to the degradation of misfolded B chain proteins. We also identified 16 proteins involved in the folding of proteins and degradation of misfolded proteins in the venom proteome (Table S3).

### Expression dynamics of mRNA encoding genes involved in protein processing

**Figure 4:**
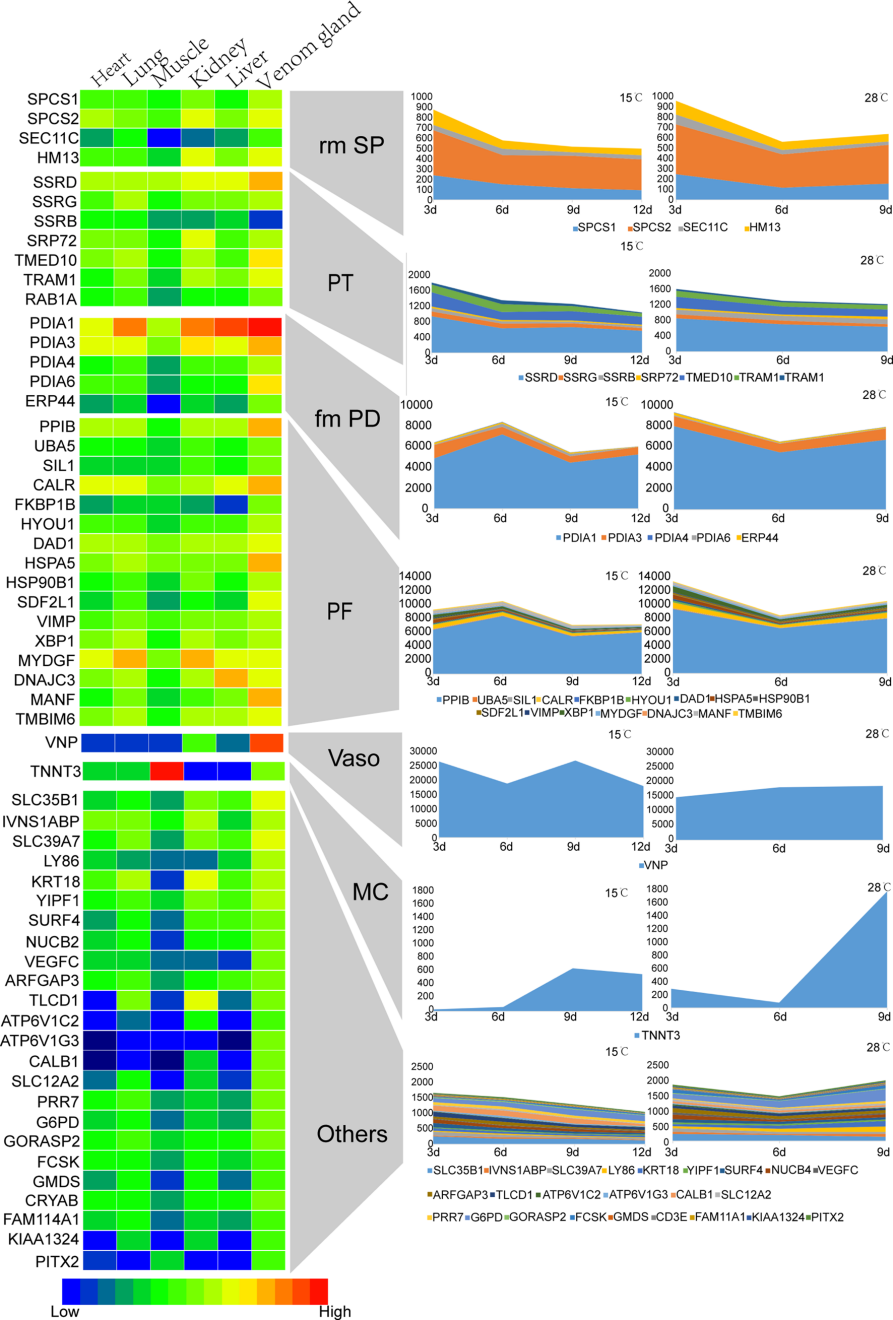
Expression dynamic of genes involved in protein processing at different replenishment times and temperatures. The expression trends in different replenishment stages and temperatures are shown in the right. *PT* protein translocation, *PF* protein folding, *Rm sp* remove signal peptide, *Fm PD* disulfide bonds, *Vaso* vasodilatation, *MC* muscle contraction. Nomenclature follows GeneCards annotations.

Snake venom glands are specialized secretory glands that contain abundant secretory cells for rapid re-synthesis of venom proteins and other components to replenish venom stores after a bite. The expression dynamics of toxin processing genes represent important markers of venom protein maturity. Therefore, 59 genes with the function above were identified and divided into seven classes: protein translocation, protein folding, removal of signal peptide, disulfide bond, vasodilatation, muscle contraction, and others (Fig. 4). These genes are likely to play an important part in the rapid synthesis, folding, and misfolding degradation of venom proteins. Several genes have been reported to assist in the folding of toxins and facilitate rapid diffusion of other toxins into prey tissues^22,23^.

We also investigated the expression dynamics of these genes. As in the case of snake venom, the expression dynamics of protein processing genes also peaked on days 3–6. Genes related to removal of signal peptide and protein translocation peaked day 3, and those involved in disulfide bond and protein folding peaked on day 6. The expression dynamics of VNP varied with temperature; expression levels were high as early as day 3 at 15 °C, but peaked at day 9 for both temperatures. In general, expression levels of protein processing genes were higher at 28 °C than at 15 °C.

In this study, TNNT3 was first found in the venom gland tissue, and only trace amounts were present on day 6 after venom expulsion, but levels increased significantly on day 9 and remained relatively stable thereafter. In viperid and elapid snakes, large striated compressor muscles attach directly to the thick connective tissue capsule of the venom glands; contraction of these muscles raises intraglandular pressure and results in rapid ejection of venom^24,25^. Fast skeletal muscle troponin T (TNNT3) is an important component of the troponin complex forming the calcium-sensitive molecular switch that regulates striated muscle contraction in response to modifications in intracellular concentration^26,27^. This suggests that although the expression level of the main toxin peaks between days 3 and 6, the formation of the mature venom gland system would take at least 9 days.

### Identification of positive selection genes (PSGs) among protein processing genes

**Figure 5:**
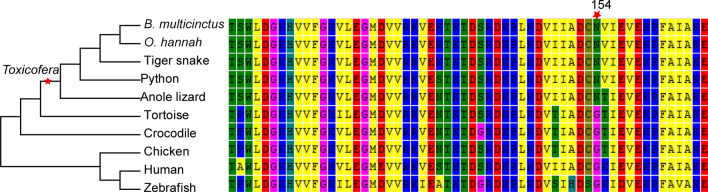
Maximum-Likelihood tree of the PPIB gene family, positive selection clade and site was marked by red pentagram. Snakes and a clade of lizards was referred to “Toxicofera”^15^.

Given the specificity of the venom gland system in snake venom synthesis, we surveyed our full-length transcriptome assemblies, as well as those available for other species, for genes involved in protein processing. A phylogenetic approach was constructed and Ka/Ks was calculated by pamlX. Unexpectedly, these highly efficient genes had never undergone positively selection in snakes. Interestingly, the PPIB gene appeared to be under positive selection in Toxicofera (posterior probability 0.979; p < 0.01) (Fig. 5). A common view is that venom system evolved a single time in the common ancestor of snakes and a clade of lizards, referred to collectively as the Toxicofera^28^.

## Discussion

### PacBio full-length transcriptome

Since the coming on of the second-generation sequencing technologies, our understanding of transcriptome complexity have been significantly improved by a series of RNA-seq studies. However, obtaining full-length transcripts still is a challenge because of difficulties in the short-read-based assembly. Recently, the single-molecule long-read sequencing technology from PacBio has provided a better alternative to sequencing full-length cDNA molecules; this have been successively used for whole-transcriptome profiling in human and other species^29–31^. PacBio long-read sequencing technology was also used for validating and predicting gene models in eukaryotes^32^. In comparison with short-read sequencing, PacBio sequencing has the foremost advantages of better completeness when sequencing cDNA molecules^29,33^. In the present study, we employed the PacBio single-molecule long-read sequencing technology to obtain high-quality whole-transcriptome sequencing data, enabling us to sequence pooled RNA samples from different organs or tissues with reduced sequencing cost. Those results therefore expand the reference set of gene transcriptions and provide the sequence data of the toxins in *B. multicinctus*, which would provide the reference for designation of precise treatment plans by producing polyclonal and monoclonal antibody for venomous snakebites.

### Analysis of DEGs

The results of the DEGs analysis indicated that expression levels varied with changes in replenishment time and temperature. GO and KEEG analysis indicated that the DEGs were mainly genes involved in protein processing (Table S2). However, except for some low-expressed toxins, there were no significant differences in gene expression of the rich toxins. These results indicate that the venom gland system includes complex background operations to keep venom expression relatively constant and facilitate prey capture and predator defense at any time. Genes involved in protein processing may play an important part in venom expression.

### Venom gland transcriptome and venom proteome comparison

In this study, 3FTxs and β-BGT comprised the main toxin classes in *B. multicinctus* , consistent with the results of a previous study^34^. Three new *B. multicinctus* toxin genes, metalloproteinase and hyaluronidase were identified and found to be slightly expressed in the venom gland (Fig. 1), 5′-nucleotidase had very low expression levels, with a FPKMmax < 1, and were considered not expressed. While these three toxins form the dominant component of viperid and some elapid venoms. This expression pattern suggests that these three venom genes, which originated in the ancestors of snakes and advanced snakes^35^, have been eliminated with the diversification of some elapid species.

In the venom gland transcriptome, no changes in venom composition and proportion were apparent within 12 days of replenishment. Previous work by Chen et al.^36,37^ identified mRNA as a frequent and stable component of venoms. The previous study proposed that the physiochemical properties of venom, including high concentrations of citrate^39,40^ and a weakly acidic pH^41,42^, may confer stability on venom components. However, the venom proteome varies with changes in replenishment time and temperature. The correlation between transcriptomic and proteomic abundances in snake venom has traditionally been poor^43,44,45^. A previous study proposed that expression of mRNAs and protein abundance are not necessarily identical owing to various cellular regulatory processes^46,47^. These factors may explain some of the variance in the data presented here and elsewhere. For instance, although the A and B chains of β-BGT were expressed in parallel, there was a significant bias in the A to B chain ratio in the venom proteome. In vitro folding of the β-BGT B chain commonly results in a homodimer, yet this does not occur for the A chain. 3FTxs contain eight or ten cysteine residues, but only one native fold is commonly found in *B. multicinctus* venom^48^. However, in vitro folding of 3FTxs generally results in low folding yields, and commonly accumulated misfolded or aggregated products^48^. These results indicated that appropriate folding and disulfide bond pairing of protein probably involved a variety of chaperones and other proteins participation; this is supported by that a series of relative protein have been identified in snake venoms and venom glands^48–50^. In this study, 16 proteins and 59 genes involved in the folding of proteins and degradation of misfolded proteins were identified in the venom proteome and venom gland transcriptome of *B. multicinctus* respectively (Table S4). Overall, the venom microenvironment appears to impart unusual stability to mRNA, together with variations in proteins; these observation could be exploited to significantly expand research into venoms and venom biology. The identification of genes involved in protein processing may help to produce ancillary proteins for appropriate post-translational modifications of those toxins; it has proven to be relatively difficult to produce such recombinant proteins.

### Expression dynamics of mRNA encoding genes involved in protein processing

In this study, we first investigated the time scale of venom synthesis in *B. multicinctus* and established that expression of the main venom transcripts peaks between days 3 and 6 of the cycle of protein replenishment. Re-synthesis of venom components examined in this study followed a different pattern to that reported in an earlier study^4^, with two subunits of β-BGT expressed in parallel. This result indicates that replenishment may be dependent on the biological roles of venom proteins.

Protein processing is a basic physiological process of venom, through which it forms a stable scaffold and becomes a mature venom. As in venom, protein processing-related gene expression also peaked between days 3 and 6, suggesting that these genes may have important roles in venom protein processing (Fig. 4). However, TNNT3 peaked on day 9. This suggests that the snake venom of *B. multicinctus* can only function effectively on the ninth day of venom re-synthesis. Understanding venom regeneration kinetics and the maturation time of the venom gland system will help to design venom extraction protocols that can maximize the yield and quality of the collected venoms. In addition, PPIB under positive selection and had a high expression level in the venom gland in *B. multicinctus* venom (Figs. 4, 5). Further experiments are required to verify the role of PPIB in assisting oxidative folding of toxin proteins.

## Materials and methods

### Venom samples and standards

The 39 specimens were adult *B. multicinctus* captured in Julong Artificial Breeding and Farming Center for Special Economic Animals, Liu an, Anhui Province, China. Venom was removed by inducing the snakes to bite through a plastic film stretched over a strile container, while the snakes was fed at three different temperatures (15, 28, 32 °C) respectively. Then, three snakes were euthanized with a single-step sodium pentobarbital (100 mg/kg) injection at four time ports (3, 6, 9, 12 days) respectively. The rest of three snakes was fed more than 25 days and euthanized as the above method. After euthanized, integrated venom gland was swiftly removed with scalpel and milk the venom into microfuge tubes, lyophilized and stored at − 80 °C until analyzed. Venom gland tissues were collected according to the protocol described by Tan et al.^44^. The venom gland was sectioned into dimensions of < 5 × 5 mm and preserved in RNAlatersolution at a 1:10 volume ratio, then stored at 4 °C overnight and transferred to − 80 °C for further analyzed.

### Library construction and PacBio sequencing

Total RNA was extracted from six pooled tissues with TRIzol reagent (Life Technologies, Carlsbad, USA) according to the manufacturer’s instructions. The concentration and quality of RNA were determined using a NanoDrop spectrophotometer. The integrity of the RNA was examined with an Agilent 2100 device. A SMARTer PCR cDNA synthesis kit (Clontech, Palo Alto, CA, USA) was used to synthesize first-strand cDNA according to the manufacturer’s instructions. After subsequent PCR amplification, quality control, and purification, a library was constructed using the cDNA products and a PacBio SMRTbell template prep kit. The library was quantified by Qubit 2.0, and Agilent 2100 was used to evaluate its size. Finally, the library was subjected to full-length transcriptome sequencing on the PacBio RS II platform by Beijing Biomarker Technologies Co., Ltd (Beijing, China).

### Transcriptome assembly

Full-length transcriptomes were acquired through a three-step process. In the first step, raw reads were processed into CSSs using RS_reads of an insert with SMRT Portal, and FLNC transcripts were identified by searching CCSs for polyA tail signals and 5′ and 3′ cDNA primers. In the second step, CCS sequences were corrected by next-generation sequencing using LSC software^51^. Third, CD-HIT^52^ was used to merge highly similar sequences and remove redundant sequences from the high-quality transcripts to yield unigenes for subsequent analysis.

### Transcriptome sequencing of venom gland

The mRNA of 29 venom glands was prepared with TRIzol reagent, and RNA-seq libraries were constructed using the Illumina mRNA-seq prep kit. Briefly, mRNA molecules were gathered using oligo (dT) magnetic beads. A fragmentation buffer was used to further fragment the mRNA, and random hexamers were used as primers during the first-strand cDNA synthesis. After double-stranded cDNA obtained, a QiaQuick PCR kit (Qiagen, Valencia, CA, USA) was used to purify the cDNA. Sequencing was performed using the Illumina platform (Illumina, San Diego, CA, USA). Raw reads were filtered and trimmed using the Trimmomatic package^53^, and clean reads were mapped to the full-length transcriptome using HISAT2^54^. Subsequently, StringTie was used to construct transcripts and estimate their expression^55^. Gene expression was measured as FPKM. TheDEseq2^55^ was used to compute differences in the significance of transcript abundance. Fold Change ≥ 2.00 and Adjusted P value ≤ 0.05 were considered to indicate DEGs. Differential expression analysis was carried out at the gene level. Heatmaps were constructed using Heml. Stacked charts, pie charts, and line charts were constructed using Excel.

### Proteome quantification and analysis

First, 100 µg of venom protein samples was placed in a centrifuge tube with 5 μL 1 M DTT and incubated at 37 °C for 1 h. Then, 20 μL indole-3-acetic acid (IAA) was added at room temperature and the tube was kept in the dark for 1 h. Samples were centrifuged and the supernatant was removed. After addition of 100 μL UA, samples were centrifuged and the supernatant was removed again, after which 100 μL 50 mM NH_4_HCO_3_, was added, followed by centrifugation and removal of the supernatant once more. Trypsin was added at a 50:1 protein/enzyme ratio, followed by hydrolysis at 37 ºC for more than 12 h for liquid chromatography with tandem mass spectrometry analysis.

Second, the samples were separated using high-pressure liquid chromatography (Ultimate 3000, Thermo Scientific, USA) with a C18 trap column (C18 1.9 m 0.15 × 120 mm). The gradient was composed of 0.1% methanoic acid (A) and 0.1% methanoic acid/80% acetonitrile (B), and the linear gradient was set as follows: 0–8 min for 94–91% A, 8–24 min for 91–86% A, 24–60 min for 86–68% A, 60–75 min for 68–32% A, and 75–80 min for 32–5% A. The flow rate was 600 nL.min^-1^. Finally, each separated sample was analyzed with a Q-Exactive HF spectrometer (Thermo Scientific, USA); see Fig. S16 for parameters. The acquired datasets (.raw files) were analyzed using MaxQuant and the built-in Andromeda search engine against the UniProt database.

### Identification of PSGs

The CDS sequence of *B. multicinctus* and nine other species—*Notechis scutatus* (tiger snake), *Python bivittatus* (python)*, Anolis carolinensis* (anole lizard), *Ophiophagus hannah*, *Chelonoidis abingdonii* (tortoise), *Crocodylus porosus* (crocodile), *Homo sapiens* (human), *Danio rerio* (zebrafish), and *Gallus* (chicken)—were retrieved for alignment using MEGA7. The branch-site model of CODEML in PAML 4.7 was used to test for potential PSGs, with *B. multicinctus*, elapid snakes, snakes, or Toxicofera set as the foreground branch and the others as background branches. The genes with corrected P-value < 0.05 and containing at least one positively selected site (posterior probability ≥ 0.90, empirical Bayes analysis) were defined as PSGs.

### Ethics statement

*B. multicinctus* is a common reptile species and is not included in the List of Endangered and Protected Animals in China. All experiments were performed in accordance with the proposals of the Committee for Research and Ethical Issues of the International Association for the Study of Pain and were approved by the Animal Ethics Committee at the Chengdu University of Traditional Chinese Medicine.

## Supplementary information

Supplementary Information.

## Data Availability

All primary data are archived and accessible at the NCBI and supplementary material. Accession number: PRJNA608620.
