# Supplementary material for: Kinetic analysis of effects of temperature and time on the regulation of venom expression in Bungarus multicinctus
Source: Sci Rep. 2020 Aug 24;10:14142. doi: 10.1038/s41598-020-70565-2 (PMC7445180; doi:10.1038/s41598-020-70565-2)
Supplement: Supplementary file 1 — Supplementary Information. [file 41598_2020_70565_MOESM1_ESM.docx]

**Supplementary informations**

**Kinetic Analysis of temperature and time effect on the regulation of venom expression in *Bungarus multicinctus***

Xianmei Yin^1^, Shuai Guo^1^, Jihai Gao^1^, Lu Luo^2^, Xuejiao Liao^1^, Mingqian Li^3^, He Su^4^, Zhihai Huang^4^, Jiang Xu^2^, Jin Pei^1^, Shilin Chen^2^

^1^Pharmacy College, Chengdu University of Traditional Chinese Medicine, Key Laboratory of Distinctive Chinese Medicine Resources in Southwest China, Chengdu 611137, PR China

^2^ Key Laboratory of Beijing for Identification and Safety Evaluation of Chinese Medicine, Institution of Chinese Materia Medica, China Academy of Chinese Medical Sciences, Beijing 100700, China

^3^ Cancer Institute of Integrated traditional Chinese and Western Medicine, Zhejiang Academy of Traditional Chinese Medicine, Tongde hospital of Zhejiang Province, Hangzhou, Zhejiang, 310012, China;

^4^ The second Clinical College of Guangzhou university of Chinese Medicine, Guangzhou 510006, China.

Correspondence: jxu@icmm.ac.cn (J.X.); [peixjin@163.com](mailto:peixjin@163.com) (J.P); slchen@icmm.ac.cn (S.C.).

Xianmei Yin, Shuai Guo and Jihai Gao contributed equally to this work.

**Supplementary Figures**


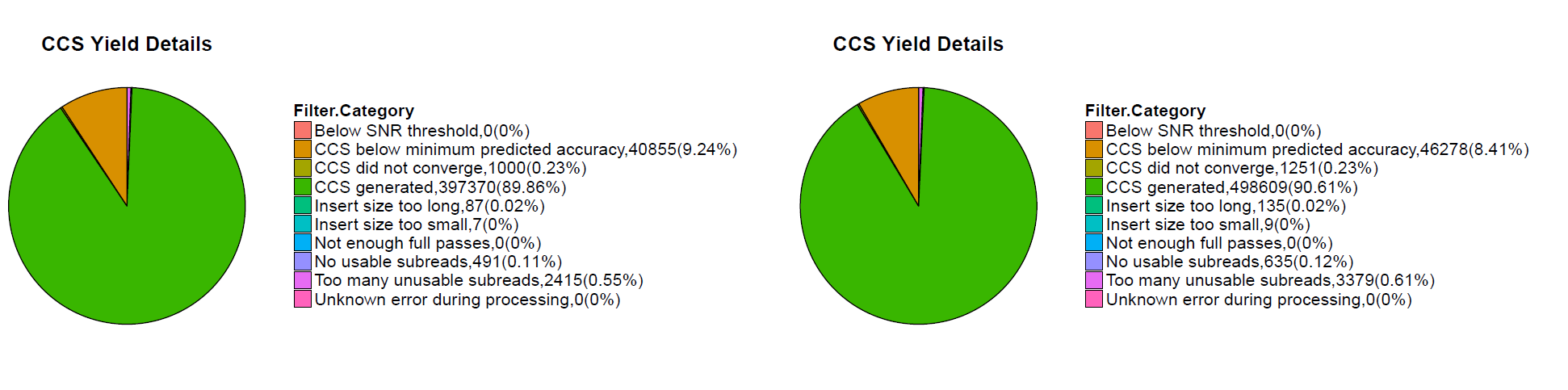


Figure S1. Circular consensus sequence (CCS) filter statistics.


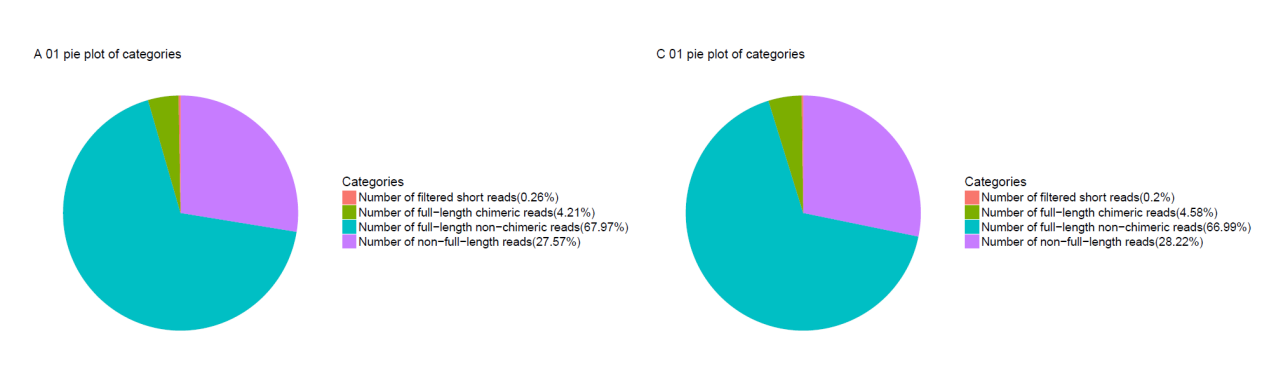


Figure S2. Circular consensus sequence (CCS) classification.


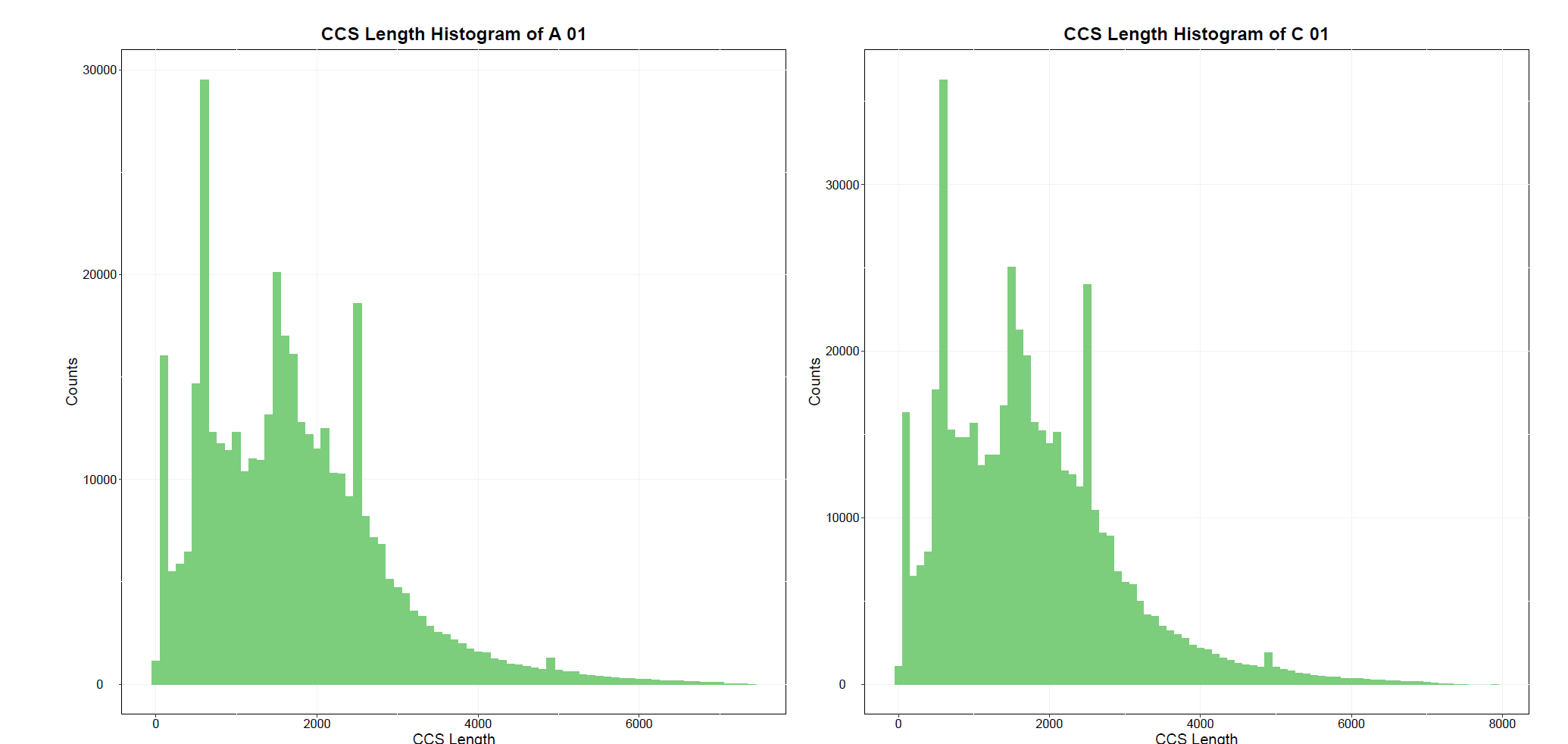


Figure S3. CCS sequence length distribution after correction.


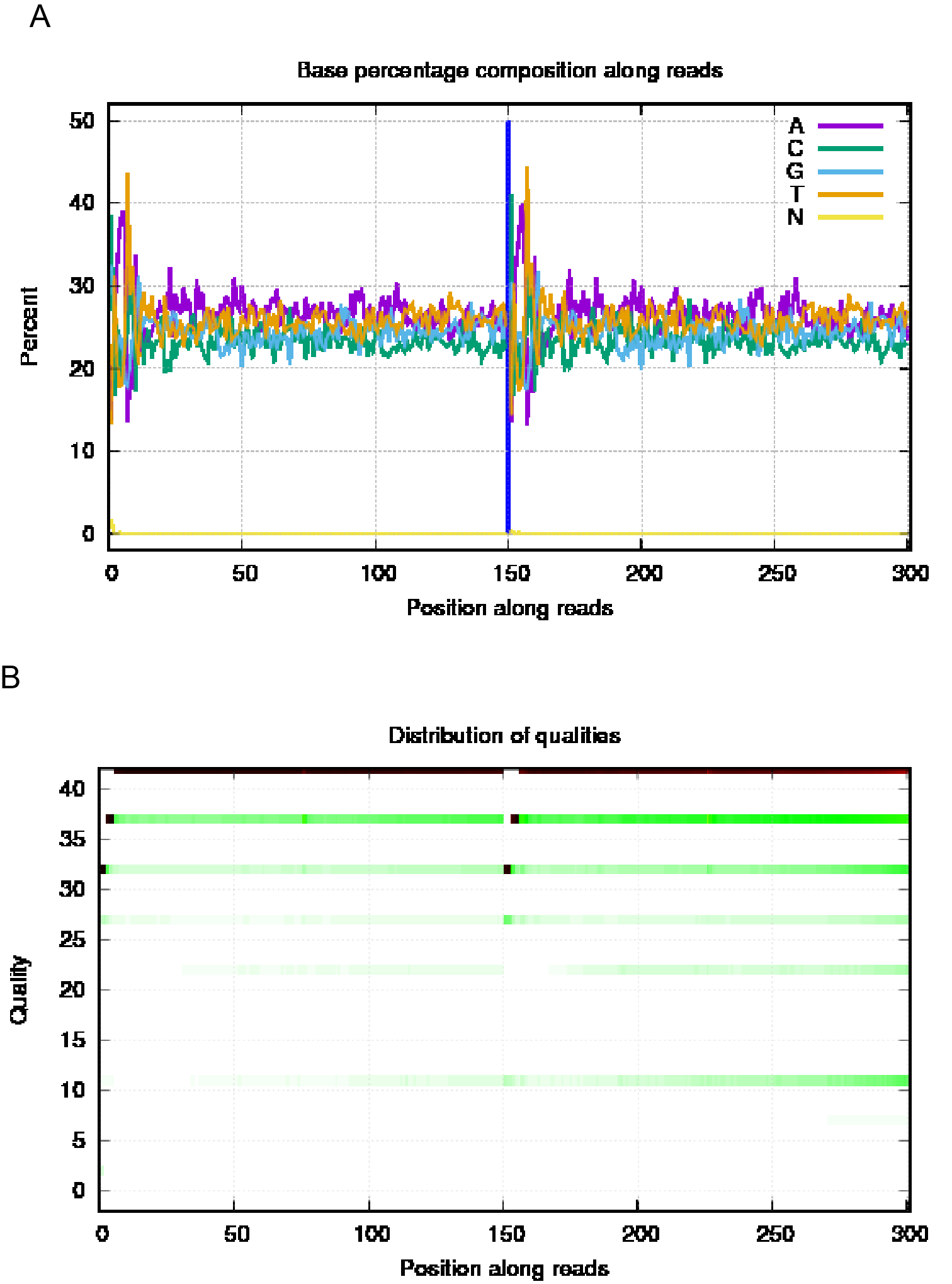


Supplementary figure 4. (A) Distribution of base composition on clean reads. X axis represents base position along reads. Y axis represents base content percentage. (B) Distribution of base quality on clean reads. X axis represents base positions along reads. Y axis represents base quality value.


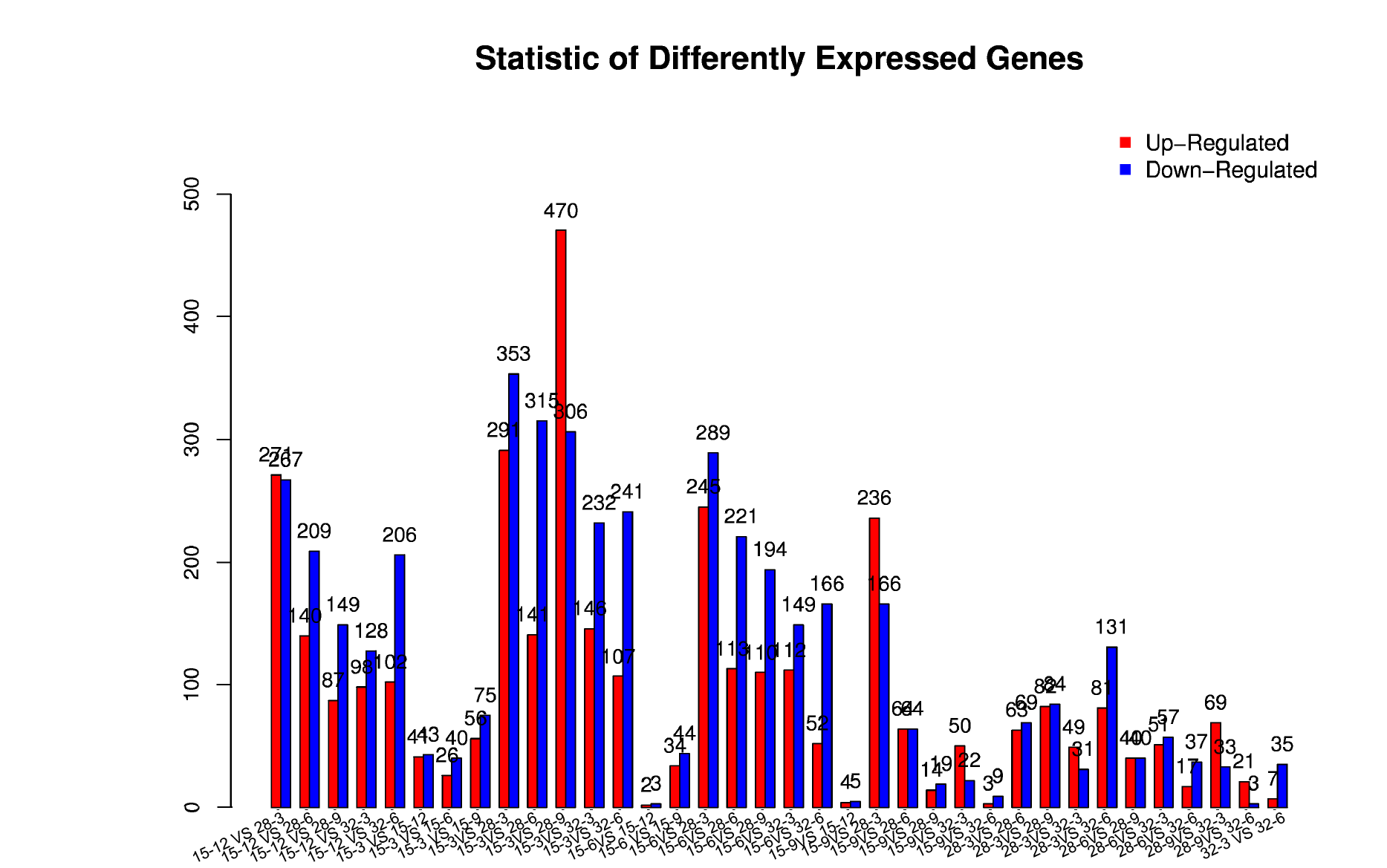


Supplementary figure 5. Summary of DEGs. The x-axis represents the compared samples.The y-axis represents DEG numbers. Red color represents up-regulated DEGs. Blue color represents down-regulated DEGs.


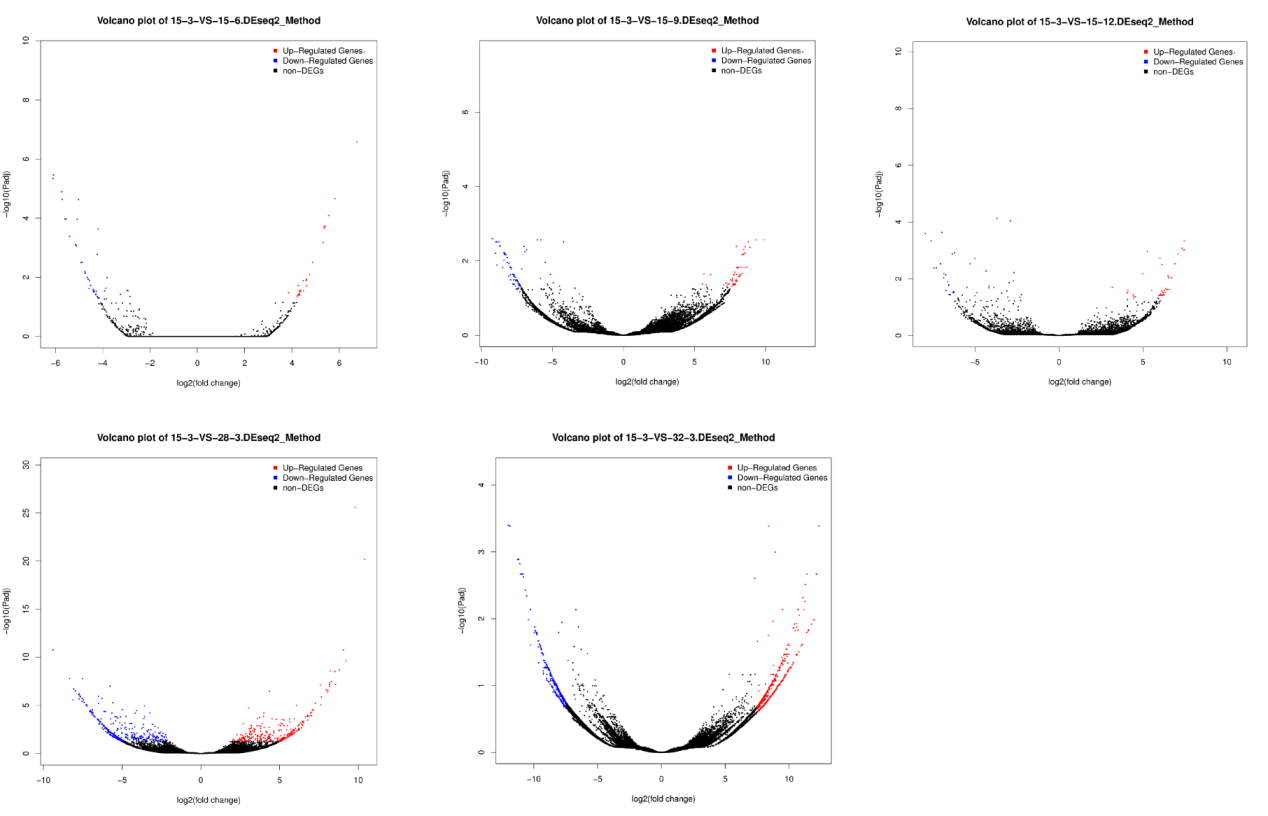


Supplementary figure 6. Volcano plot of DEGs. The x-axis represents the log2-transformed fold change. The y-axis represents the -log10-transformed significance.


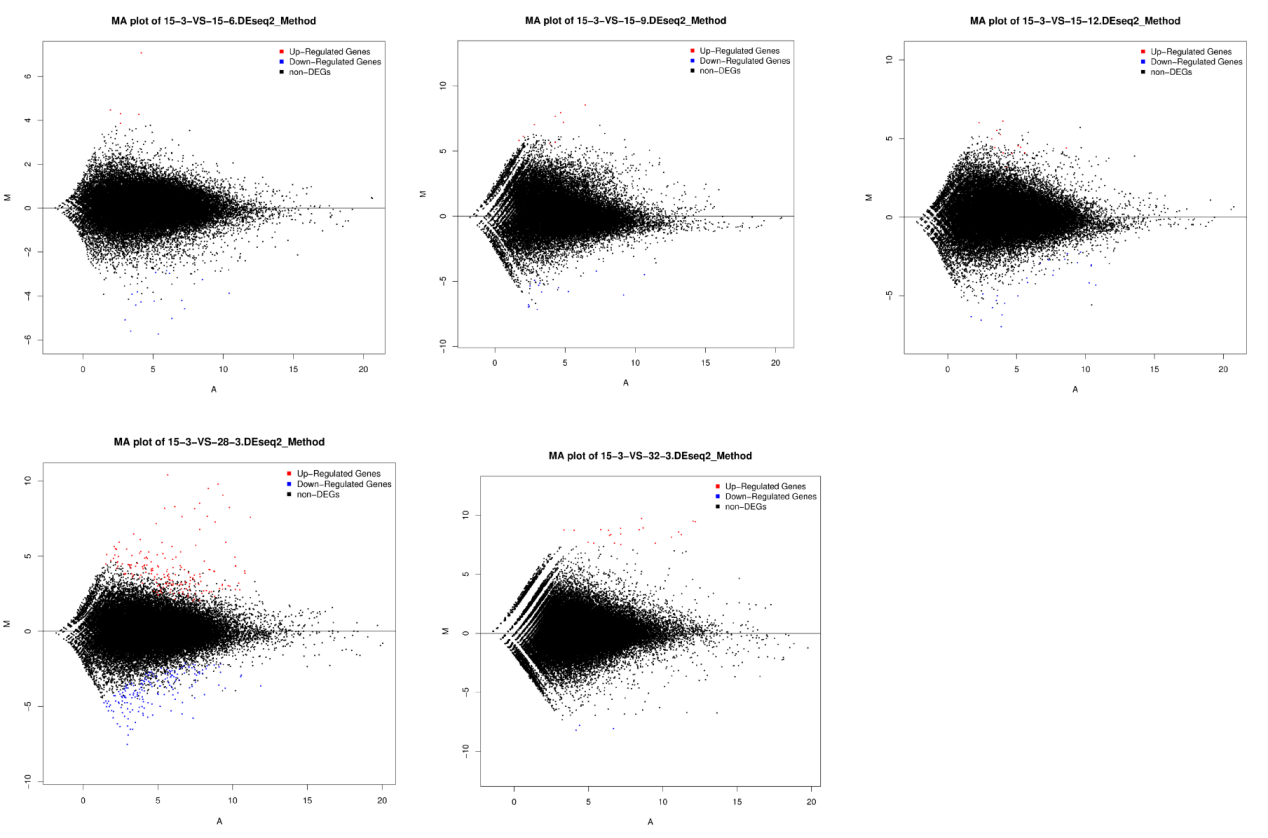


Supplementary figure 7. Minus-versus-add (MA) plot of DEGs. The x-axis represents value A (log2-transformed mean expression level). The y-axis represents value M (log2-transformed fold change). Red point represent up regulated DEGs, Blue color represent down regulated DEGs.


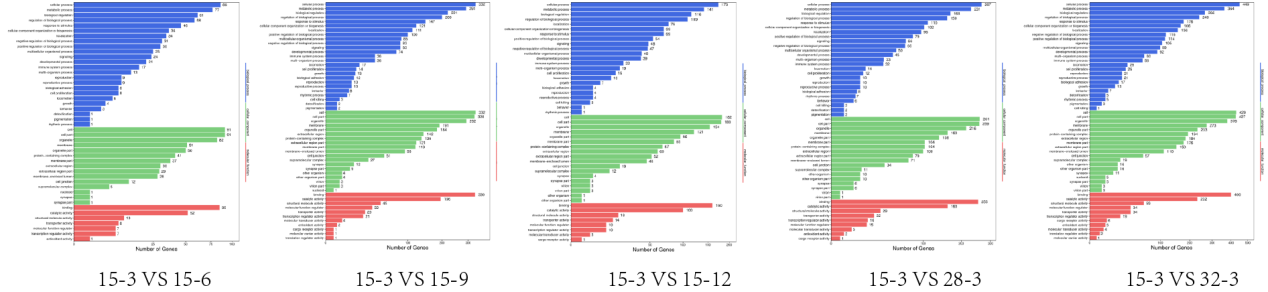


Supplementary figure 8. Gene ontology (GO) classification of DEGs. The y-axis represents the GO term. The x-axis represents the number of DEGs.


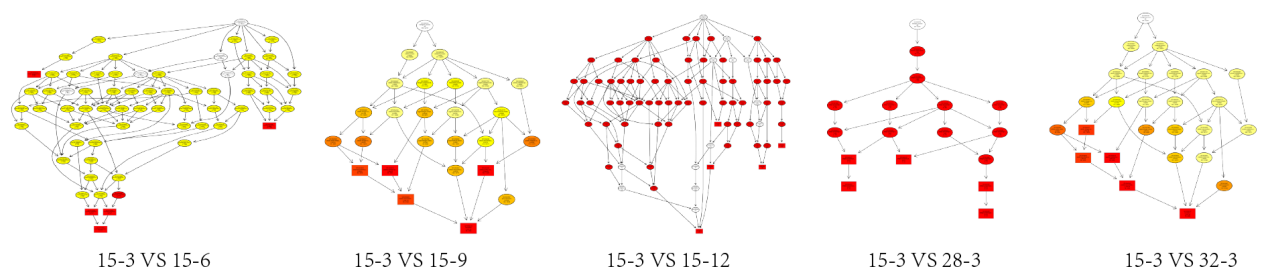


Supplementary figure 9. Biological Process gene ontology (GO) classification of DAGs.


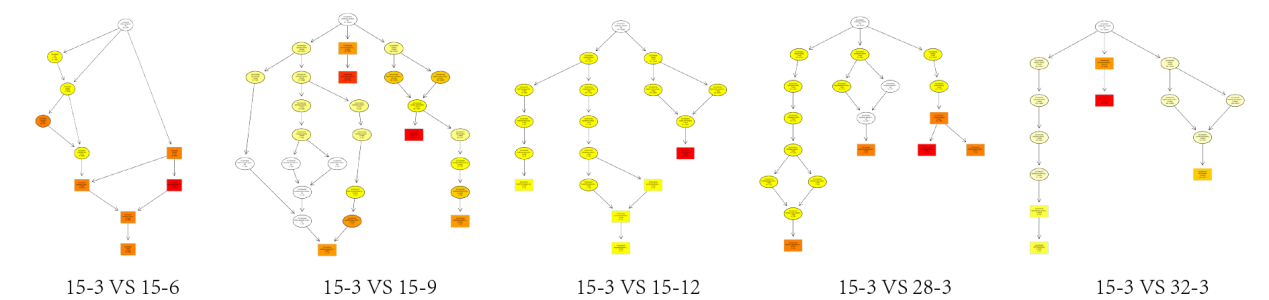


Supplementary figure 10. Molecular gene ontology (GO) classification of DAGs


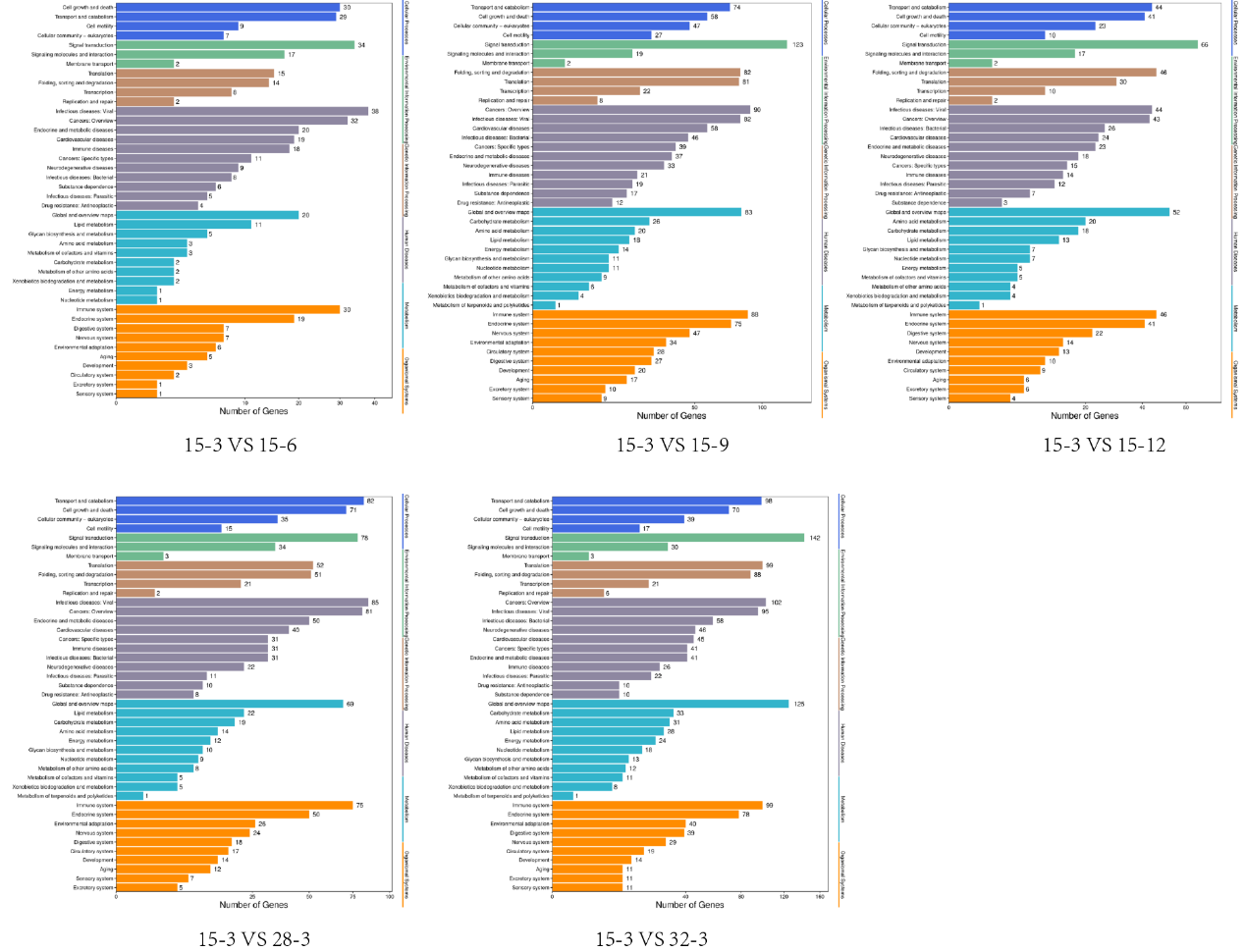


Supplementary figure 11. Pathway classification of diﬀerentially expressed genes (DEGs). The x-axis represents the number of DEGs. The y-axis represents the pathway name.


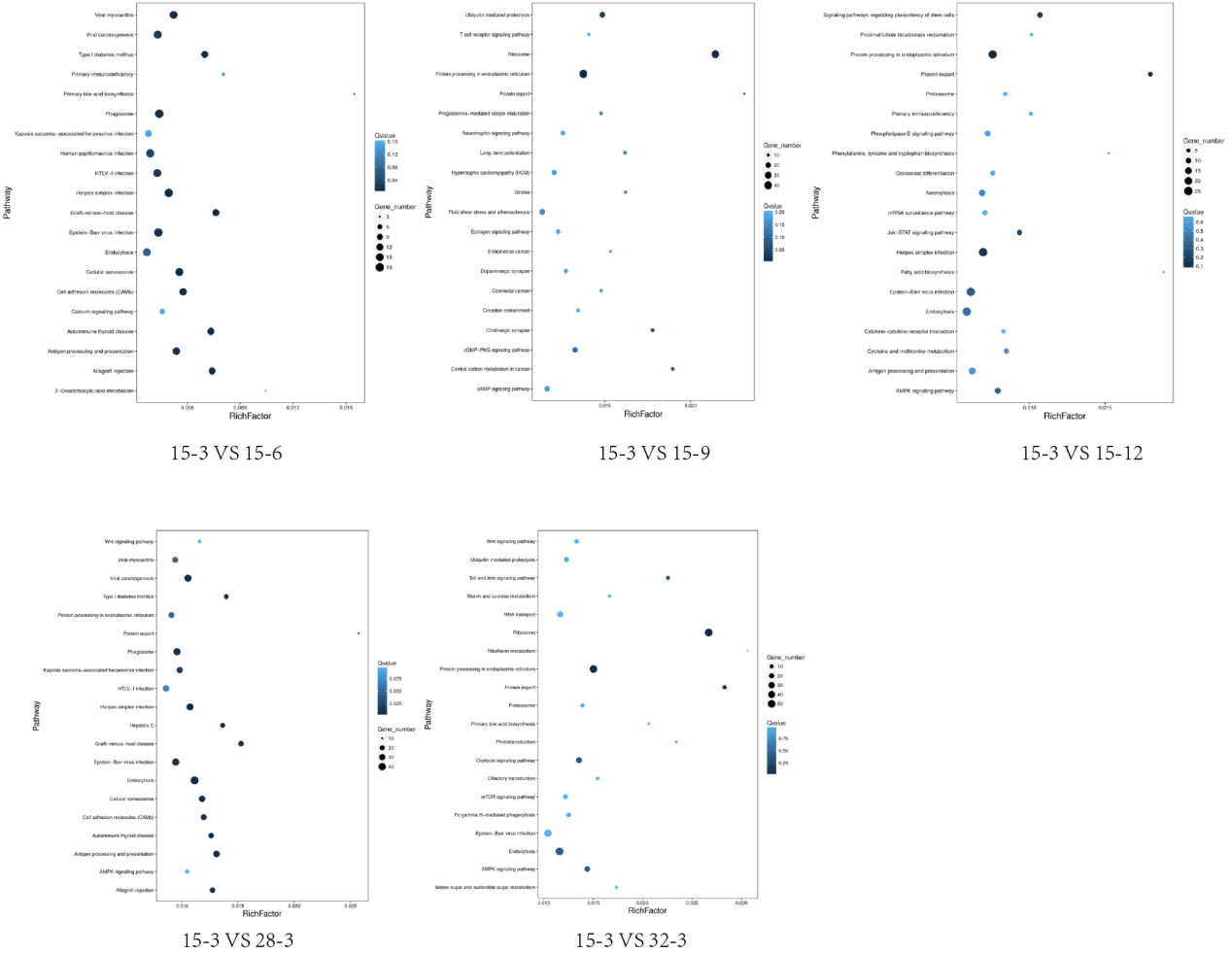


Supplementary figure 12. Pathway classification of diﬀerentially expressed genes (DEGs). The x-axis represents the rich factor. The y-axis represents the pathway name.


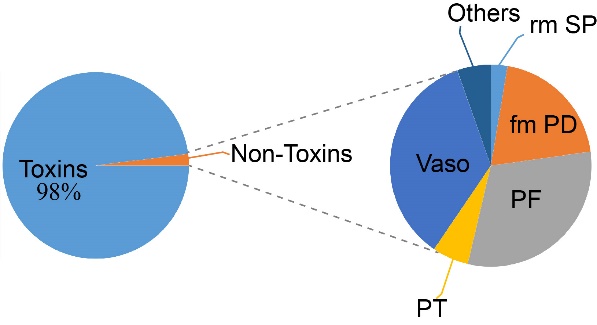


Supplementary figure 13. Toxin genes proportion of venom gland transcription. Toxin genes constitute >98% of transcriptome’s expression, protein processing genes constitute >95% of redundant transcript


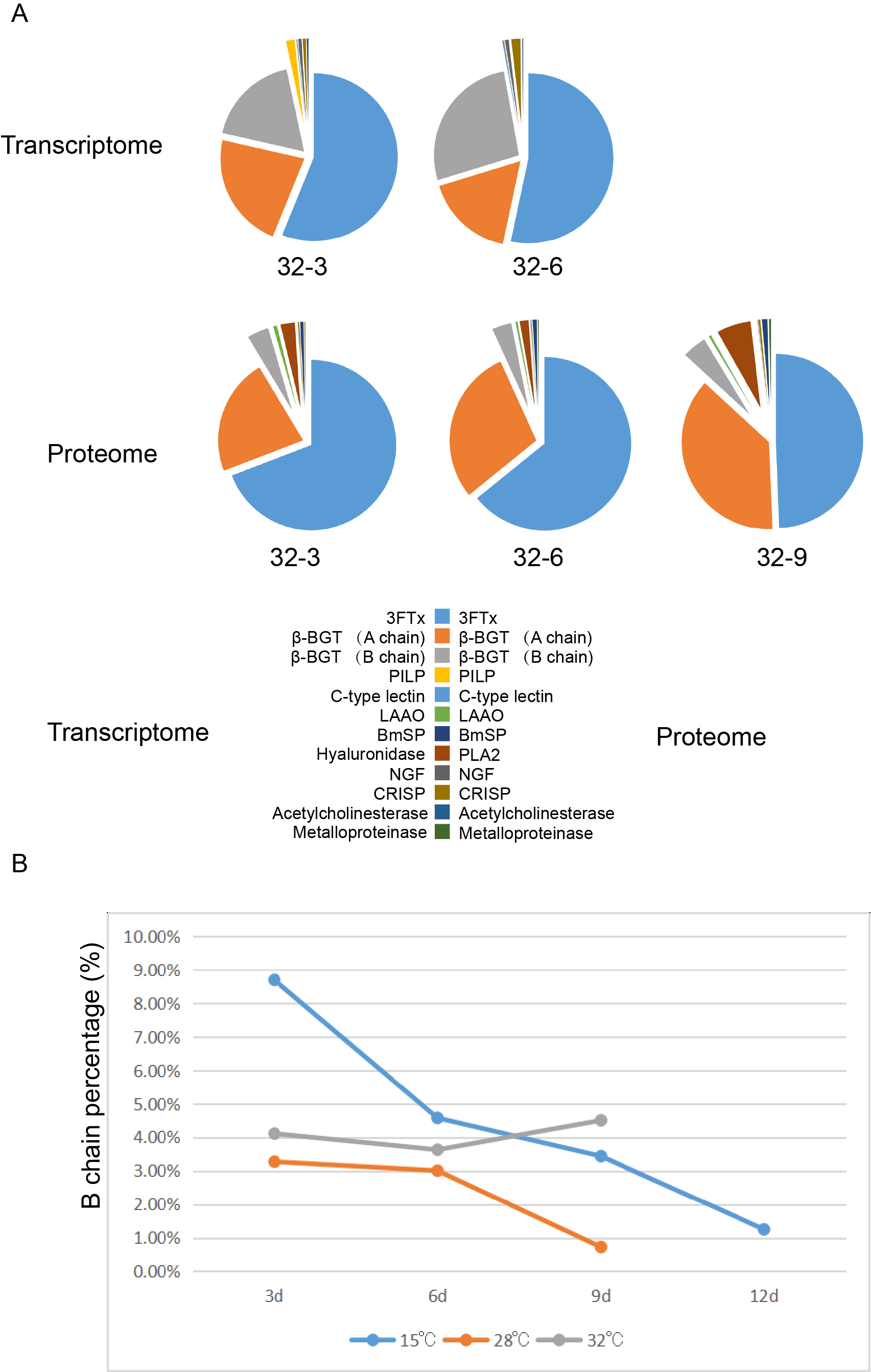


Supplementary figure 14. (A) Distribution of toxins in the venom gland transcriptomes and venom proteomes of *B. multicinctus* at different replenishment time and temperature. (B) The proportion of venom B chain of β-BGT in the venom proteome at different replenishment time and temperature.


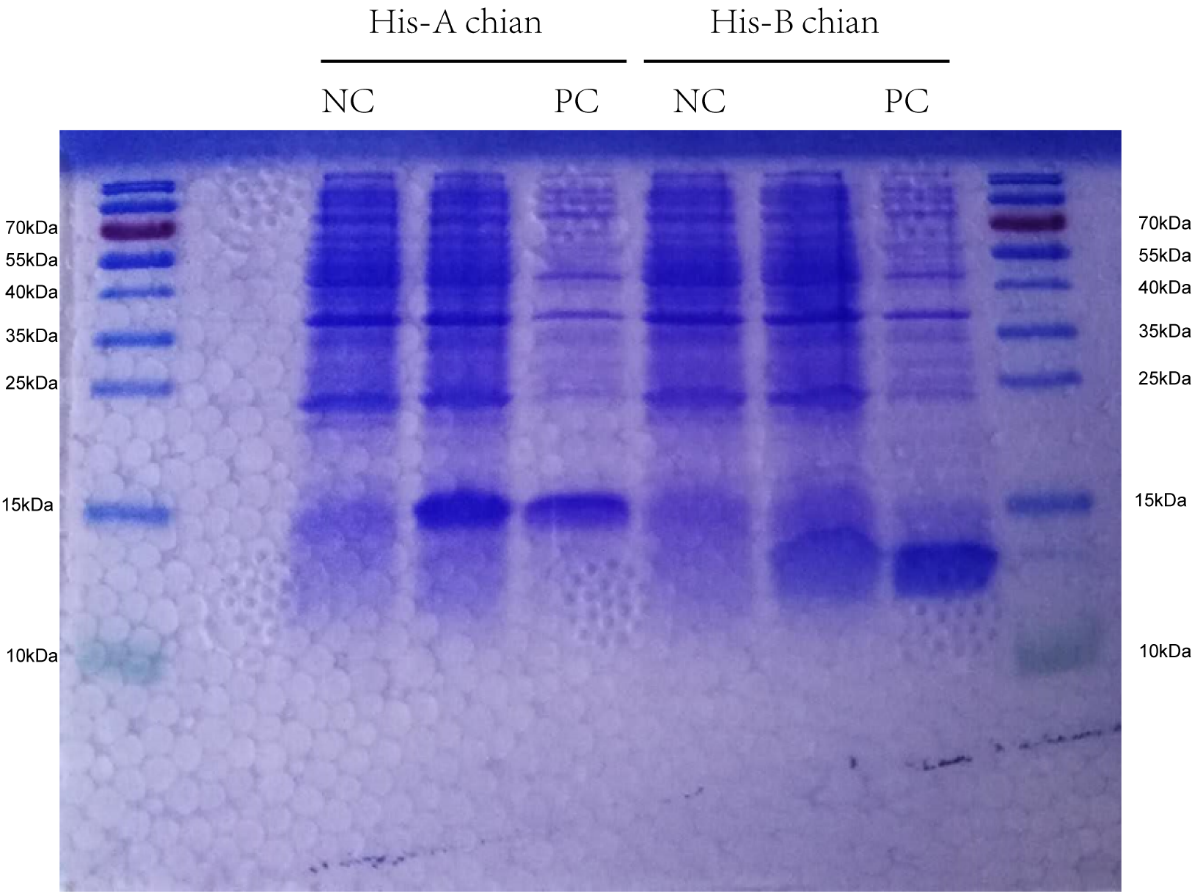


Supplementary figure 15. SDS-PAGE show the size of His6-B chain and His6-A chain expressed in *E. coli.*


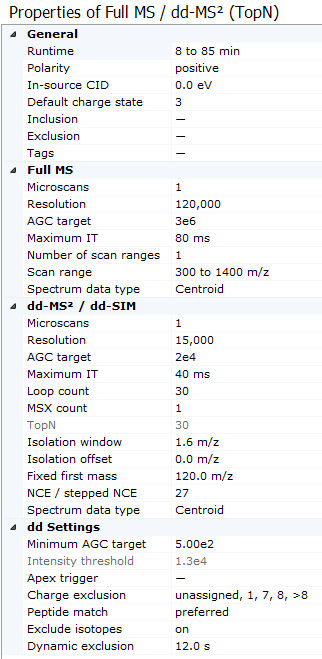


Supplementary figure 16. Q-Exactive HF parameters

**Supplementary tables**

Table S1. Summary of sequence reads for 29 RNA samples including three replicate control treatments.

| **Sample** | **Clean Reads(M)** | **Clean Bases(Gb)** | **Q20(%)** | **Q30(%)** | **GC(%)** | **Read Length(bp)** | **Total CleanReads** | **Total MappingRatio** | **Total GeneNumber** |
| --- | --- | --- | --- | --- | --- | --- | --- | --- | --- |
| B0-0-1 | 48.8663M | 7.3299 | 97.99 | 95.39 | 47.79 | 150 | 48866280 | 77.25% | 33214 |
| B0-0-2 | 48.7411M | 7.3112 | 98.24 | 95.48 | 45.7 | 150 | 48741110 | 82.01% | 28922 |
| B0-0-3 | 48.0772M | 7.2116 | 98.05 | 95.54 | 47.2 | 150 | 48077180 | 78.51% | 33446 |
| B15-3-1 | 48.1371M | 7.2206 | 98.07 | 95.55 | 46.71 | 150 | 48137092 | 82.04% | 31639 |
| B15-3-2 | 48.2837M | 7.2426 | 97.93 | 95.29 | 47.02 | 150 | 48283680 | 77.16% | 37816 |
| B15-3-3 | 48.4762M | 7.2714 | 97.86 | 95.14 | 47.08 | 150 | 48476162 | 76.29% | 36181 |
| B15-6-1 | 48.7990M | 7.3199 | 98 | 95.4 | 46.97 | 150 | 48799008 | 83.07% | 35228 |
| B15-6-2 | 48.2870M | 7.243 | 97.87 | 95.2 | 47.48 | 150 | 48286996 | 80.67% | 32716 |
| B15-6-3 | 48.1569M | 7.2235 | 97.96 | 94.93 | 42.86 | 150 | 48156900 | 55.46% | 39153 |
| B15-9-1 | 48.9342M | 7.3401 | 97.74 | 94.66 | 42.86 | 150 | 48934250 | 35.36% | 24215 |
| B15-9-2 | 48.6908M | 7.3036 | 97.91 | 95.29 | 47.09 | 150 | 48690780 | 83.02% | 32502 |
| B15-9-3 | 48.1589M | 7.2238 | 98.16 | 95.31 | 45.64 | 150 | 48158898 | 66.83% | 34575 |
| B15-12-1 | 48.4143M | 7.2622 | 97.95 | 95.36 | 47.11 | 150 | 48414346 | 81.98% | 33683 |
| B15-12-2 | 48.6722M | 7.3008 | 97.87 | 94.86 | 45.01 | 150 | 48672206 | 45.45% | 20914 |
| B15-12-3 | 48.3083M | 7.2462 | 98.18 | 95.26 | 47.4 | 150 | 48308302 | 75.11% | 37654 |
| B28-3-1 | 49.1141M | 7.3671 | 98.04 | 95.46 | 46.35 | 150 | 49114112 | 74.93% | 33765 |
| B28-3-2 | 49.1738M | 7.3761 | 98.11 | 95.1 | 46.96 | 150 | 49173800 | 77.74% | 34555 |
| B28-3-3 | 49.0143M | 7.3521 | 98.04 | 95.02 | 45.89 | 150 | 49014254 | 62.55% | 35085 |
| B28-6-1 | 49.1336M | 7.37 | 98.28 | 95.48 | 47.59 | 150 | 49133628 | 78.74% | 33076 |
| B28-6-2 | 48.8851M | 7.3328 | 98.27 | 95.55 | 45.52 | 150 | 48885124 | 66.02% | 36870 |
| B28-6-3 | 48.1642M | 7.2246 | 98.04 | 95.53 | 46.72 | 150 | 48164226 | 83.32% | 33374 |
| B28-9-1 | 48.4517M | 7.2678 | 97.99 | 95.18 | 43.7 | 150 | 48451700 | 45.69% | 22136 |
| B28-9-2 | 48.6729M | 7.3009 | 98.27 | 95.49 | 46.76 | 150 | 48672922 | 77.93% | 36969 |
| B28-9-3 | 48.0723M | 7.2108 | 97.94 | 94.87 | 45.09 | 150 | 48072284 | 41.60% | 35409 |
| B32-3-2 | 45.3014M | 6.7952 | 97.93 | 94.72 | 43.38 | 150 | 45301358 | 72.16% | 31717 |
| B32-3-3 | 49.1192M | 7.3679 | 98.15 | 95.18 | 46.83 | 150 | 49119190 | 72.42% | 36065 |
| B32-6-1 | 49.3294M | 7.3994 | 98.2 | 95.38 | 46.59 | 150 | 49329448 | 63.14% | 31309 |
| B32-6-2 | 48.8672M | 7.3301 | 98.05 | 95.52 | 46.23 | 150 | 48867158 | 82.10% | 33148 |
| B32-6-3 | 49.1235M | 7.3685 | 97.51 | 94.2 | 41.17 | 150 | 49123542 | 33.53% | 20070 |

Table S2. Top ten differential gene enrichments of KEGG pathway enrichment analysis.

| **Pathway level1** | **Pathway level2** | **Average number of Genes** | **Proportion** |
| --- | --- | --- | --- |
| Environmental Information Processing | Signal transduction | 88.6 | 0.075340136 |
| Metabolism | Global and overview maps | 69.8 | 0.059353741 |
| Human Diseases | Cancers: Overview | 69.6 | 0.059183673 |
| Human Diseases | Infectious diseases: Viral | 68.8 | 0.058503401 |
| Organismal Systems | Immune system | 67.6 | 0.057482993 |
| Cellular Processes | Transport and catabolism | 65.4 | 0.055612245 |
| Genetic Information Processing | Folding, sorting and degradation | 56.2 | 0.047789116 |
| Genetic Information Processing | Translation | 55.4 | 0.047108844 |
| Cellular Processes | Cell growth and death | 54 | 0.045918367 |
| Organismal Systems | Endocrine system | 52.6 | 0.044727891 |

Supplementary table 3. Label-free quantitative proteome

| **Venom toxins** | | **proteins processing** |  |
| --- | --- | --- | --- |
| **Family** | **Toxin names** | **Protein IDs** | **Protein names** |
| 3FTx | Alpha-bungarotoxin-1 | PPIB | Peptidyl-prolyl cis-trans isomerase B |
|  | Kappa-bungarotoxin-5 | [HSPA5](https://www.genecards.org/cgi-bin/carddisp.pl?gene=HSPA5&keywords=GRP78) | Heat Shock Protein Family A (Hsp70) Member 5 |
|  | Gamma-bungarotoxin | PDIA1 | Protein disulfide-isomerase |
|  | Kappa-bungarotoxin-4 | HSP | Heat shock cognate 71 kDa |
|  | Kappa-bungarotoxin-2 | ERP44 | Endoplasmic reticulum resident protein 44 |
|  | Kappa-bungarotoxin-3 | [UGGT1](https://www.genecards.org/cgi-bin/carddisp.pl?gene=UGGT1&keywords=UDP-glucose:glycoprotein,glucosyltransferase,1) | UDP-glucose:glycoprotein glucosyltransferase 1 |
|  | BM10-1 | CALR | Calreticulin |
|  | Short neurotoxin-1 | EF2 | Elongation factor 2 |
|  | Alpha-bungarotoxin-2 | HSP90B | Heat shock protein HSP 90-beta |
|  | Short neurotoxin-1 | MANF | Mesencephalic astrocyte-derived neurotrophic factor |
|  | BM14 | Hsp90b1 | Endoplasmin |
|  | Cardiotoxin | CATB | Cathepsin B |
|  | Short neurotoxin-2 | ARF3 | ADP-ribosylation factor 3 |
|  | Alpha-bungarotoxin-3 | TERA | Transitional endoplasmic reticulum ATPase |
|  | Alpha-bungarotoxin-4 | UBE2L3 | Ubiquitin-conjugating enzyme E2 L3 |
|  | Kappa-bungarotoxin-1 | CATB | Cathepsin B |
| PLA2 | β-BGT A1 chain |  |  |
|  | β-BGT A2 chain-1 |  |  |
|  | β-BGT A3 chain |  |  |
|  | β-BGT A chain-1 |  |  |
|  | β-BGT A chain-2 |  |  |
|  | β-BGT A2 chain-2 |  |  |
|  | β-BGT A1 chain-1 |  |  |
|  | β-BGT A1 chain-2 |  |  |
|  | β-BGT A-AL2 chain |  |  |
|  | β-BGT A7 chain |  |  |
|  | Phospholipase A2 KPA2 |  |  |
|  | Phospholipase A2 |  |  |
| Kunitz | β-BGT B5-B chain |  |  |
|  | β-BGT B4 chain |  |  |
|  | β-BGT B2 chain |  |  |
|  | C-type lectin-1 |  |  |
|  | C-type lectin-2 |  |  |
|  | PILP-3 |  |  |
|  | BmSP |  |  |
|  | Serine protease inhibitor |  |  |
| LAAO | L-amino-acid oxidase |  |  |
| NGF | Venom nerve growth factor |  |  |
| CRISP | Cysteine-rich venom protein |  |  |
| Acetylcholinesterase | Acetylcholinesterase |  |  |
| Metalloproteinase | Metalloproteinase |  |  |
|  | Metalloproteinase |  |  |

Supplementary table 4. The cDNA sequence and protein molecular weight of A Chain and B chain.

| **Gene name** | **cDNA** | **Protein molecular weight (kDa)** |
| --- | --- | --- |
| A chain | GCCAACATTCCTCCCCATCCTCTCAACCTTATAAACTTCATGGAGATGATTCGATATACCATCCCCTGCGAAAAAACATGGGGTGAATATGCGGACTACGGTTGCTACTGCGGCGCAGGAGGTAGCGGGAGACCGATAGATGCCTTGGATAGGTGCTGCTATGTTCATGACAACTGCTATGGTGACGCCGAAAAAAAACATAAATGCAACCCCAAAACGCAGTCATATTCATACAAATTGACTAAACGCACGATCATCTGCTATGGTGCCGCAGGTACTTGTGGACGTATTGTCTGTGATTGTGACCGCACGGCAGCCCTCTGCTTCGGCAATTCCGAATACATCGAGGGGCACAAGAATATCGACACCGCGAGATTTTGCCAATGA | 14.33 |
| B chian | GGAAACGTCATCCGGATTGTGATAAGCCTCCTGACACCAAAATCTGTCAAACCGTTGTACGTGCCTTCTACTACAAGCCATCTGCAAAACGTTGCGTACAGTTTAGATATGGTGGCTGTAATGGCAATGGCAACCATTTCAAAAGCGACCATTTATGCCGCTGCGAGTGTCTTGAGTATCGC | 7.2 |
